# m6A-regulated tumor glycolysis: new advances in epigenetics and metabolism

**DOI:** 10.1186/s12943-023-01841-8

**Published:** 2023-08-15

**Authors:** Shi-Wei Yue, Hai-Ling Liu, Hong-Fei Su, Chu Luo, Hui-Fang Liang, Bi-Xiang Zhang, Wei Zhang

**Affiliations:** 1 Hepatic Surgery Center, Tongji Hospital, Tongji Medical College, Huazhong University of Science and Technology, Wuhan, China; 2 Hubei Key Laboratory of Hepato‑Pancreatic‑Biliary Diseases, Tongji Hospital, Tongji Medical College, Huazhong University of Science and Technology, Wuhan, China; 3 Clinical Medical Research Center of Hepatic Surgery at Hubei Province, Wuhan, China

**Keywords:** m6A, Tumor, Glycolysis, Tumor therapy

## Abstract

Glycolytic reprogramming is one of the most important features of cancer and plays an integral role in the progression of cancer. In cancer cells, changes in glucose metabolism meet the needs of self-proliferation, angiogenesis and lymphangiogenesis, metastasis, and also affect the immune escape, prognosis evaluation and therapeutic effect of cancer. The n6-methyladenosine (m6A) modification of RNA is widespread in eukaryotic cells. Dynamic and reversible m6A modifications are widely involved in the regulation of cancer stem cell renewal and differentiation, tumor therapy resistance, tumor microenvironment, tumor immune escape, and tumor metabolism. Lately, more and more evidences show that m6A modification can affect the glycolysis process of tumors in a variety of ways to regulate the biological behavior of tumors. In this review, we discussed the role of glycolysis in tumor genesis and development, and elaborated in detail the profound impact of m6A modification on different tumor by regulating glycolysis. We believe that m6A modified glycolysis has great significance and potential for tumor treatment.

## Introduction

Cancer cells have the potential to replicate indefinitely, which is inseparable from a continuous supply of energy and the support of macromolecular raw materials. In order to maintain their proliferative ability, cancer cells alter their metabolic state through various pathways. This makes the metabolism of tumor cells significantly different from that of normal cells [1]. As early as 1920s, Warburg and his colleagues found a special phenomenon in the process of glycolysis of cancer cells: even in the presence of oxygen, cancer cells mainly rely on glycolysis to provide energy. This is known as the Warburg effect [2]. Until 2011, the concept of cancer metabolism reprogramming, as one of the main characteristics of cancer, has attracted widespread attention [3]. Tumor cells’ metabolic reprogramming primarily involves glucose, fatty acid, amino acid, and mitochondrial metabolism. Among these, glycolysis, situated at the core of the tumor cell metabolic network, plays an irreplaceable role in tumor initiation and progression. For example, during the proliferation of tumor cells, glycolysis can provide enough energy and metabolic intermediates to ensure the smooth progress of cell biosynthesis [4]. The lactate produced by glycolysis of tumor cells has also been proved to inhibit the function of various immune cells, and is also related to the resistance of tumor immunotherapy [5, 6]. Given the crucial role of tumor glycolysis in its genesis, development, and treatment, it is necessary to explore the glycolysis of tumor cells at a deeper level.

Methylation of m6A is an epigenetic regulatory mechanism that has become increasingly familiar in recent years, and is also a widespread RNA modification in mammalian eukaryotic cells [7]. The so-called m6A modification is a methylation substitution reaction, which occurs at the N6-position of adenosine, and is most common in the 3’UTR region and RRACH ([G > A] m6AC [U > A > C]) sequence of RNA [8–10]. This dynamic reversible process is regulated by the interaction between the “writers” (methyltransferases that catalyzes methylation), the “erasers” (demethylases that removes methylation) and the “readers” (RNA-binding proteins that recognizes methylation) [11]. In tumor cells, m6A modification is widely involved in gene expression by regulating processes such as RNA splicing, maturation, stability, translation, and localization, thus controlling the progression of cancer [12]. Compelling evidence indicates that m6A regulates tumor metabolism through multiple pathways, significantly impacting tumorigenesis and tumor development [13, 14]. For example, m6A modifications can modulate fatty acid metabolic enzymes and pathways, thereby regulating lipid metabolism in tumor cells [15, 16]. Additionally, by influencing glutamine metabolism, a pivotal component of tumor amino acid metabolism, m6A modification can indirectly affect nucleotide synthesis [17, 18]. Moreover, m6A participates in tumor chemotherapy resistance by regulating mitochondrial metabolism [19]. However, beyond the aforementioned metabolic pathways, the most prominent area of research on m6A modification in tumor metabolism is tumor glycolysis. Over the past few years, studies on m6A in tumor glycolysis have experienced explosive growth. Numerous investigations have unveiled the molecular mechanisms through which m6A modification regulates tumor glycolysis and its pivotal role in this metabolic process.

In this review, we provide a brief introduction to the regulation of m6A modification. Then, we systematically describe the effects of glycolysis on tumor proliferation, angiogenesis and lymphangiogenesis, metastasis, immune escape, prognosis evaluation and treatment. Finally, we analyze the potential mechanism by which m6A affects the development of various tumors through the regulation of glycolysis, providing a theoretical basis for the future targeting of m6A modification and glycolysis for tumor treatment.

## The m6A modification

**Fig. 1:**
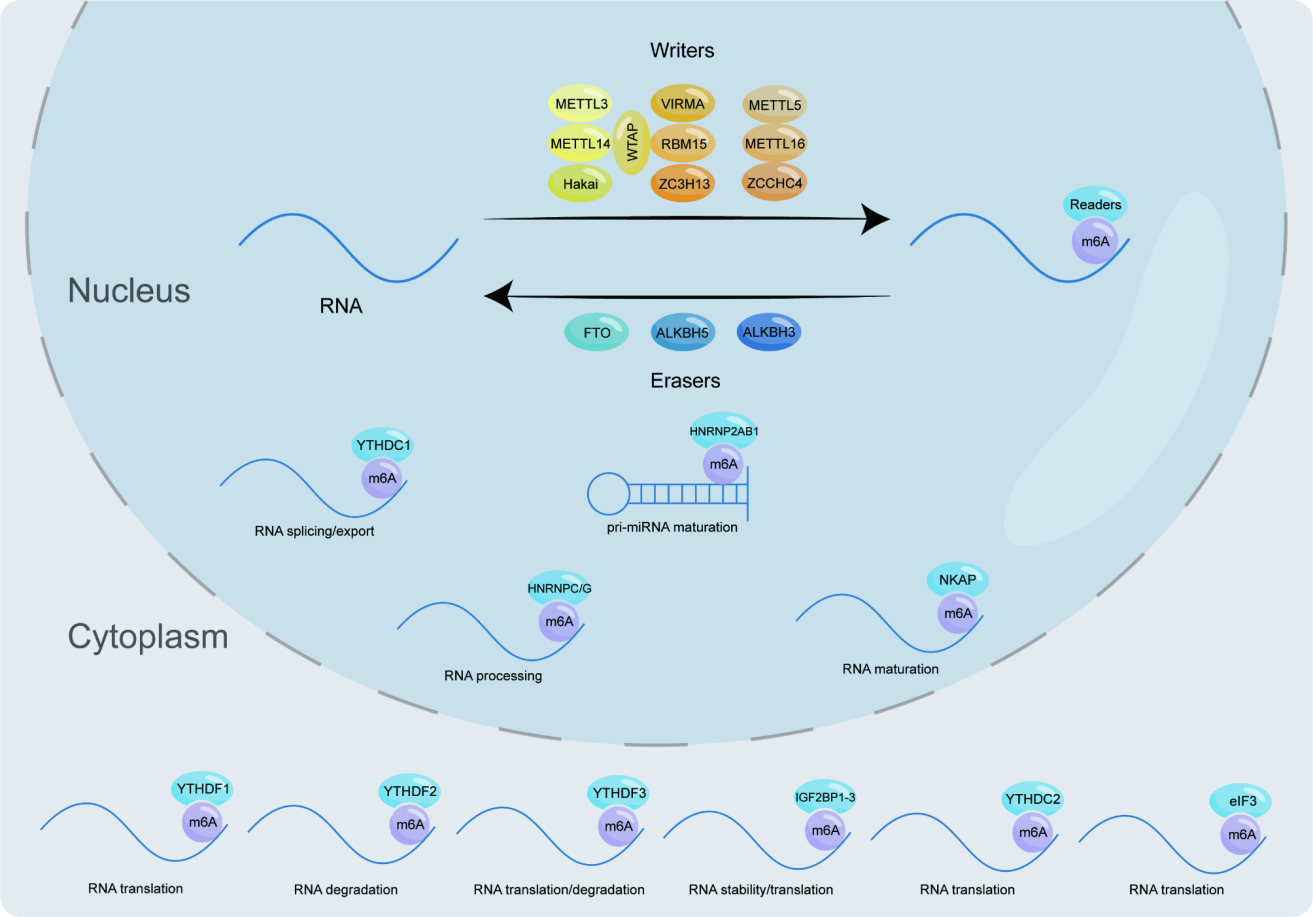
Regulation of m6A modification. The writers and erasers of m6A are responsible for regulating the methylation level of related RNA. Then, various m6A readers can recognize and combine with m6A modified RNA to regulate gene expression. METTL3, Methyltransferase-like 3; METTL14, Methyltransferase-like 14; WTAP, Wilms tumor 1-associated protein; RBM15, RNA binding motif protein 15; VIRMA, Vir-like m6A methyltransferase associated; ZC3H13, zinc finger CCCH-type containing 13; METTL16, Methyltransferase-like 16; METTL5, Methyltransferase-like 5; ZCCHC4, zinc finger CCHC-type containing 4; FTO, Fat mass and obesity-associated protein; ALKBH3, ALKB homologue 3; ALKBH5, ALKB homologue 5; YTHDF1, YTH N6-Methyladenosine RNA Binding Protein 1; YTHDF2, YTH N6-Methyladenosine RNA Binding Protein 2; YTHDF3, YTH N6-Methyladenosine RNA Binding Protein 3; YTHDC1, YTH Domain Containing 1; YTHDC2, YTH Domain Containing 2; HNRNPA2B1, Heterogeneous nuclear ribonucleoprotein A2B1; HNRNPAC/G, Heterogeneous nuclear ribonucleoprotein C/G; HNRNPR, Heterogeneous nuclear ribonucleoprotein R; IGF2BP1-3, Insulin Like Growth Factor Binding Protein 1–3; NKAP, NF-κB activating protein; eIF3, Eukaryotic translation initiation factor 3

m6A modification has been identified as the most common chemical modification in eukaryotic cell mRNA and lncRNA. It is almost involved in the whole process of RNA metabolism, such as RNA processing, nuclear export, translation and degradation [20–23]. The m6A modification of RNA is dynamic and reversible. This complex reaction cannot be separated from the interaction of the “writers”, “erasers” and “readers”. Specifically, the “writers” and “erasers” of m6A are responsible for regulating the methylation level of related RNA. Then, various m6A “readers” can recognize and combine with m6A modified RNA to regulate gene expression (Fig. 1; Table 1).

**Table 1:** The function of m6A methylation enzymes in RNA metabolism

Type	Regulator	Function	Ref
Writers	METTL3	Catalyzes m6A methylation	[26]
	METTL14	Recognizes target RNAs and stabilizes METTL3	[29]
	WTAP	Regulatory subunit of m6A methyltransferase	[31]
	RBM15	Recruits METTL3 and WTAP to specific RNA sites	[33]
	VIRMA	Locates the m6A methyltransferase complex near the 3’UTR and stop codon regions of RNAs	[34]
	Hakai	Maintains the stability of m6A methyltransferase complex	[35]
	ZC3H13	Anchors WTAP in the nucleus to enhance m6A modification	[36]
	METTL16	Catalyzes m6A methylation	[38]
	METTL5	Induce the m6A methylation of 18 S rRNA	[42]
	ZCCHC4	Induce the m6A methylation of 28 S rRNA	[43]
Erasers	FTO	Removes m6A from RNAs	[45]
	ALKBH3	Removes m6A from RNAs	[46]
	ALKBH5	Removes m6A from RNAs	[46]
Readers	YTHDF1	Promotes mRNA translation initiation	[49]
	YTHDF2	Promotes mRNA degradation	[49]
	YTHDF3	Interacts with YTHDF1 to promote mRNA translation or interacts with YTHDF2 to promote mRNA degradation	[49]
	YTHDC1	Promotes mRNA splicing and regulates RNA nuclear export	[53]
	YTHDC2	Improves the translation efficiency of target mRNA	[54]
	HNRNPA2B1	Promotes primary miRNA processing	[55]
	HNRNPC/G	Interacts with m6A-modifed mRNA	[56, 57]
	HNPNPR	Associates with m6A modification	[58]
	IGF2BP1-3	Promotes the stability and translation of mRNA	[59]
	NKAP	Promotes the splicing and maturation of mRNA	[60]
	eIF3	Promotes mRNA translation	[61]

### m6A writers

As the m6A “writer”, the methyltransferase complex is composed of multiple components, which are mutually supportive and jointly regulate the methylation of nucleic acid. As is known to all, METTL3 and METTL14 are S-adenosylmethionine (SAM) dependent methyltransferase from the same family. The heterodimer formed by them plays a vital role in the regulation of m6A and is also related to the repair of DNA damage in vitro [24, 25]. Here, we mainly discuss the role of METTL3 and METTL14 as m6A “writers”. Wang et al. showed that that METTL3 folded to form a positively charged groove at the molecular level and contained a large amount of SAM, while no SAM was found in METTL14 [26]. This shows that METTL3 is the core force of catalytic methylation modification, but its catalytic ability is inhibited after the modification of small ubiquitin-like modifier on the lysine residue of METTL3 [27]. Besides, the function of METTL3 is also inseparable from the structural basis of Cys-Cys-Cys-His (CCCH)-type zinc-binding motif [28]. As another component of METTL3-METTL14 dimer complex, METTL14 can combine with RNA through its terminal RGG repeat sequence, and can also form a wide range of connections with METTL3 at the molecular level to improve the biological activity of METTL3 [26, 29]. METTL14 can also avoid the ubiquitination degradation mediated by U-box-containing protein 1 under the protection of METTL3 [30]. Wilms tumor 1-associated protein (WTAP) is one of the components of the core complex that catalyzes the modification of m6A. It exists as a regulatory subunit of METTL3-METTL14 complex, and its expression is also regulated by METTL3 [31, 32]. RNA binding motif protein 15 (RBM15) is mainly used to bind with METTL3 and WTAP. It also improves the nucleating efficiency of mRNA by promoting the binding of RNA helicase DBP5 with mRNA [33]. VIRMA plays a role in recruiting the catalytic core complex composed of METTL3, METTL14 and WTAP. It mainly plays its catalytic function in the 3’UTR region of RNA and near the termination codon [34]. Hakai is a ring finger E3 ubiquitin ligase, which plays an irreplaceable role in maintaining the stability of m6A methyltransferase complex [35]. ZC3H13, like a rivet, connects RBM15, WTAP and VIRMA, and is also related to the nuclear positioning of WTAP, VIRMA and Hakai [36, 37].

In addition to the above mentioned, there are also some methyltransferases that cannot be ignored. For example, METTL16, which is a methyltransferase gradually known in recent years, regulates the expression of MAT2A mRNA and U6 snRNA through its N-terminal domain and C-terminal VCRs, maintains the homeostasis of SAM in cells, and is also related to the repair of DNA after damage [38–41]. Moreover, METTL5 and ZCCHC4 are responsible for the methylation of 18 S rRNA and 28 S rRNA in cells [42, 43]. The specific mechanism of their function needs further study.

### m6A erasers

Fat mass and obesity-associated protein (FTO), ALKB homolog 5 (ALKBH5) and ALKB homolog 3 (ALKBH3) are all members of the dioxygenase ALKB protein family. They rely on α-ketoglutarate and iron (II) to reduce the level of m6A modification of RNA [44, 45]. At the microstructure level, ALKBH3, ALKBH5 and FTO all have shallow gaps, so they prefer to combine with single-stranded nucleic acids such as mRNA [46]. Besides, the demethylation activity of FTO is affected by the sequence and tertiary structure of RNA, and it also has SFPQ (an RNA-binding protein) as its chaperone protein. SFPQ can combine with the CUGUG sequence on RNA and recruit FTO to promote proximal demethylation [47, 48].

### m6A readers

Unlike “writers” and “erasers”, m6A “readers” do not directly change the methylation level, but recognize and combine the methylation sites of RNA. In this process, various “readers” combine with various m6A sites, affecting the fate of RNA.

Proteins YTHDF1-3 containing the YT521-B homology (YTH) domain are common “readers” of m6A modification. It is generally believed that YTHDF1 can induce mRNA translation; YTHDF2 accelerates the degradation of mRNA; YTHDF3 has the above two functions [49]. But recently, a new model of YTHDF proteins showed that YTHDF1/2/3 acted on the same subset of mRNAs, resulting in its degradation, but did not promote translation [50, 51]. The model also indicates that there is a certain functional compensation among them [52]. Another type of m6A “readers” with YTH domain are YTH Domain Containing 1 (YTHDC1) and YTHDC2, the former is related to RNA splicing and nuclear export, and the latter is related to RNA translation [53, 54]. Heterogeneous nuclear ribonucleoprotein (HNRNP) family is another m6A “reader”. Among them, heterogeneous nuclear ribonucleoprotein A2/B1 (HNRNPA2B1) can combine with miRNA containing RGm6AC sequence, recruit microRNA microprocessor complex protein DGCR8 to splice the precursor of miRNA, and promote the formation of mature miRNA [55]. Both HNRNPC and HNRNPG are related to the special mechanism of m6A switch. The so-called m6A switch refers to the process that the structure of m6A-modified RNA changes, affecting the binding of RNA-binding proteins to corresponding sites, so as to regulate the relevant life activities in cells [56, 57]. The latest research shows that HNPNPR is also a member of HNRNPs protein family, which has strong correlation with m6A modification and participates in tumor glycolysis [58]. Additionally, the insulin-like growth factor 2 mRNA-binding proteins (IGF2BPs) are mainly involved in regulating the stability and translation of mRNA [59]. NF-κB activating protein (NKAP) promotes the splicing and maturation of mRNA [60]. Eukaryotic translation initiation factor 3 (eIF3) is also considered as a “reader” of m6A and plays a central role in the initiation process of eukaryotic translation [61]. It can be seen that the role played by m6A “readers” is very complex and diverse, including but not limited to the regulation of RNA splicing, maturation, stability, translation, and localization. In this respect, there are still many unknown “readers” waiting to be discovered.

## Glycolysis process

Glycolysis is the process of decomposing one molecule of glucose into two molecules of pyruvate, and then producing two molecules of ATP [62]. In this process, extracellular glucose is transferred to the cell fluid with the help of glucose transporters (GLUT). Then glucose is decomposed into mutually convertible glyceraldehyde 3-phosphate and dihydroxyacetone phosphate under the action of hexokinase (HK), glucose-6-phosphate isomerase (GPI), phosphofructokinase-1 (PFK) and aldolase (ALD) [63–65]. So far, glucose containing six carbon atoms has been decomposed into two three-carbon units. After that, glyceraldehyde 3-phosphate is finally converted into two molecules of pyruvate under the catalysis of glyceraldehyde-3-phosphate dehydrogenase (GAPDH), phosphoglycerate kinase (PGK), phosphoglycerate mutase (PGAM), enolase (ENO) and pyruvate kinase (PK) [62, 66].

When the cell is aerobic and has mitochondria, pyruvate usually enters mitochondria for tricarboxylic acid cycle, producing a large amount of ATP to provide energy for cells; However, pyruvate is reduced to lactate if the cells are in hypoxia or lack of mitochondria [67]. In these processes, metabolic intermediates can also enter other biochemical reaction processes, so that various intracellular reactions can be coordinated and orderly. However, biochemist Otto Warburg found a special phenomenon: in cancer cells, even if they have normal mitochondria and sufficient oxygen, they rarely undergo oxidative phosphorylation (OXPHOS). On the contrary, cancer cells mainly rely on glycolysis to provide energy. This characteristic is the expression that cancer cells can tolerate hypoxic environment, and also increases the level of lactate in tumor cell microenvironment, stimulating tumor growth and development [68].

## Glycolysis and tumor

**Fig. 2:**
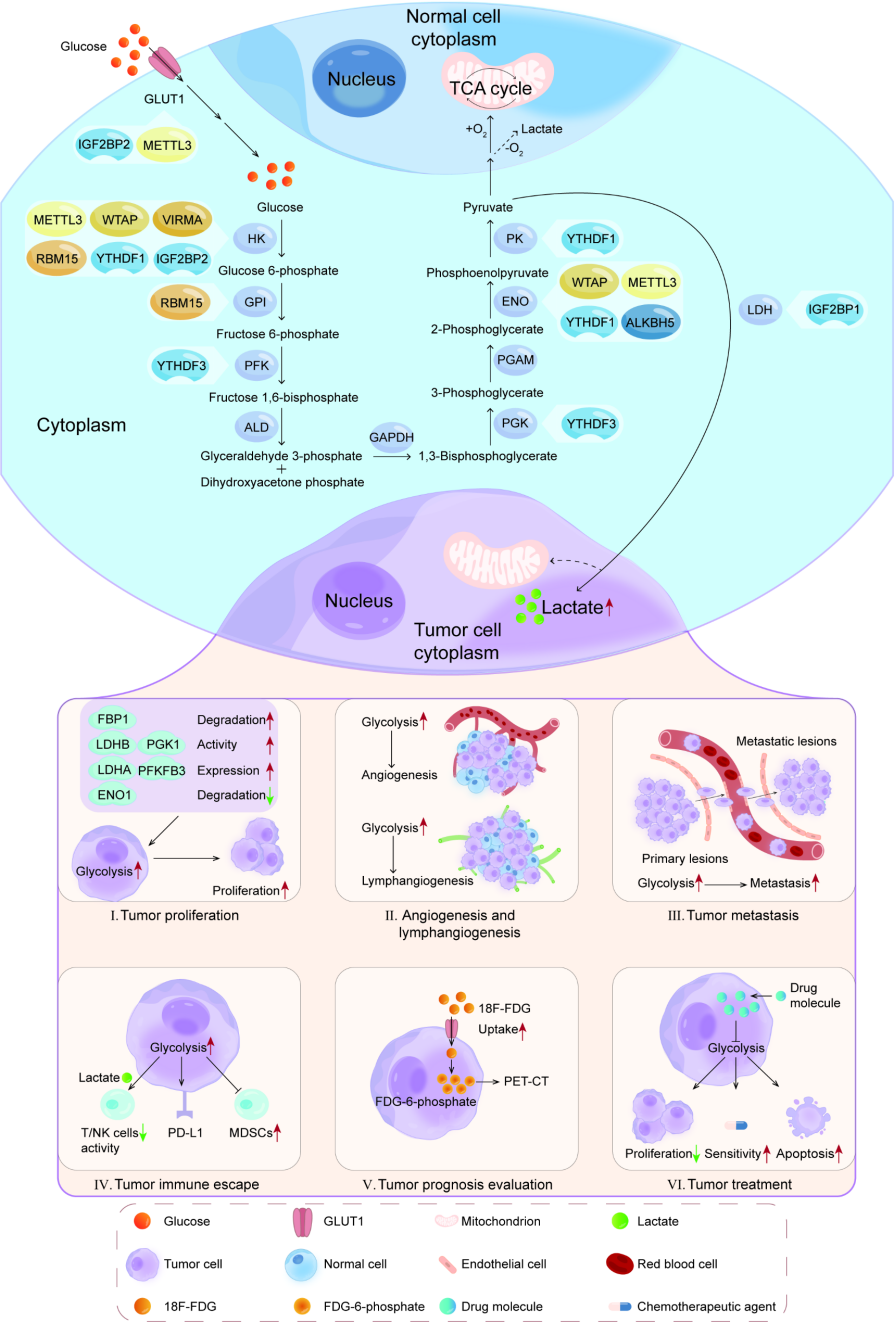
The relationship between glycolysis and tumors. In the upper part of the figure, the glycolysis pathway is depicted for both normal and tumor cells, with a focus on the involvement of m6A regulators in glucose metabolism. The lower part of the figure illustrates how glycolysis impacts various aspects of tumor biology, including proliferation, angiogenesis and lymphangiogenesis, metastasis, immune escape, and its relevance to tumor prognosis evaluation and treatment. (I) Changes in glycolysis enzymes within tumor cells influence tumor proliferation by affecting glycolysis. (II) Enhanced tumor glycolysis contributes to angiogenesis and lymphangiogenesis. (III) Elevated tumor glycolysis promotes tumor metastasis. (IV) Increased tumor glycolysis inhibits the activity of T and NK cells, affects PD-L1 expression, and promotes the generation of MDSCs, thereby inducing tumor immune escape. (V) The substantial uptake of 18 F-FDG by tumor cells leads to the accumulation of FDG-6-phosphate, which can be imaged using PET/CT. (VI) Attenuation of tumor glycolysis inhibits proliferation, enhances chemosensitivity, and promotes apoptosis in cancer cells

For most normal cells, they prefer OXPHOS to provide energy for themselves. However, in tumor cells, glycolysis holds a unique position [69]. This is because compared to OXPHOS, glycolysis produces less ATP but at a faster rate. Therefore, at the same time, glycolysis can produce more ATP to provide energy for the life activities of tumor cells [70]. Besides, various intermediate products produced during the glycolysis process are crucial for the synthesis of biological macromolecules such as lipids and nucleic acids within cells, contributing to tumor cell proliferation [71]. Lactate, one of the products of glycolysis, can effectively inhibit the function of a variety of immune cells and contribute to the immune escape of tumor cells [5]. In recent years, accumulating studies have confirmed that tumor glycolysis plays an indispensable role in tumor progression, including tumor proliferation, angiogenesis and lymphangiogenesis, metastasis, immune escape, and has a certain correlation with tumor prognosis evaluation and treatment (Fig. 2; Table 2).

**Table 2:** The role of glycolysis in the genesis and development of tumors

Classification	Mechanism	Result	Ref
Tumor proliferation	TRIM47 can ubiquitinate FBP1 and accelerate the degradation of FBP1	Promote pancreatic cancer glycolysis and proliferation	[72]
	Aurora-A phosphorylates LDHB to reduce the inhibition of substrate in the reaction	Promote tumor glycolysis and proliferation	[73]
	Increase the expression of LDHA through STAT3/LINC00671/LDHA axis	Promote thyroid cancer glycolysis and proliferation	[74]
	LINC00930 recruit the RBBP5-GCN5 complex to the PFKFB3 promoter and increase H3K4 trimethylation and H3K9 acetylation levels in the PFKFB3 promoter region	Promote nasopharyngeal carcinoma glycolysis and proliferation	[75]
	The activity of PGK1 modified by O-GlcNAcylation is improved	Promote colon cancer glycolysis and proliferation	[76]
	CD47 prevents ENO1 from ubiquitin-mediated degradation by inhibiting FBXW7	Promote colorectal cancer glycolysis and proliferation	[77]
	Increase the expression of ENO1 through circ-ENO1/miR-22-3p/ENO1 axis	Promote lung adenocarcinoma glycolysis and proliferation	[78]
Tumor angiogenesis and lymphangiogenesis	DKK2 and LRP6 cooperate to activate downstream AKT/mTOR signal pathway to accelerate aerobic glycolysis of colorectal cancer cells	Accelerate colorectal cancer angiogenesis	[80]
	S100A4 affects the position and movement of the tip cells in the new lymphatic vessels by regulating glycolysis	Accelerate melanoma lymphangiogenesis	[81]
Tumor metastasis	Oxidized ATM can phosphorylate GLUT1 and increase PKM2 expression to promote glycolysis and lactate production. Lactate can activate TGFβ1/p38 MAPK/MMP2/9 signal axis, stimulate the mitochondrial activity of cancer cells	Promote the invasion and metastasis ability of breast cancer	[84]
	CircRPN2 inhibits aerobic glycolysis of HCC by accelerating ENO1 degradation and regulating the miR-183-5p/FOXO1 axis	Inhibit the metastasis of HCC	[85]
	Through ZEB1/PFKM/glycolysis axis	Promote the tumorigenesis and intrahepatic metastasis of HCC	[86]
Tumor immune escape	Enhanced glycolysis of cancer cells inhibits T cell and NK cell activity by intensifying the accumulation of lactate	Promote lung cancer immune escape	[90]
	Glycolysis can induce the expression of PD-L1 and CTLA4	Promote breast cancer immune escape	[91]
	Glycolysis can promote the expression of G-CSF and GM-CSF in TNBC cells, and induce the generation of MDSCs	Promote TNBC immune escape	[92]
	HK2 acts as a protein kinase to phosphorylate IκBα and induce PD-L1 expression	Promote glioblastoma immune escape	[93]
	Tumor-associated macrophages secrete TNF to promote tumor glycolysis, and inhibit the expression of PD-L1 in tumor cells	Promote NSCLC immune escape	[94]
Tumor prognosis evaluation	Tumor cells are characterized by increased glucose metabolism	Evaluate the prognosis of multiple tumors	[95–100]
Tumor treatment	DHNQ reduces the expression of glycolytic enzyme genes such as PFK1 and PKM2	Inhibit the genesis and development of colorectal cancer	[102]
	Inhibition of GLUT1 can significantly reduce the glycolysis of CRPC cells	CRPC is more sensitive to the treatment of enzalutamide and reduces drug resistance	[103]
	Met can activate AMP-AMPK pathway and p53 pathway and destroy glycolysis of tumor cells. Besides, glucose oxidase reduces glucose content in tumor tissue	Inhibit the development of tumor	[104]
	Chrysin slows down the glycolysis rate of liver cancer cells	Accelerate apoptosis of tumor cells	[105]
	Piperlongumine can reduce the expression of HK2, inhibit the glycolysis of cancer cells	Induce apoptosis of NSCLC cells	[106]

### Glycolysis and tumor proliferation

The life activities of cells cannot be separated from energy. Even tenacious tumor cells cannot survive without the energy provided by glycolysis. In pancreatic cancer, TRIM47 can ubiquitinate fructose-1,6-diphosphatase (FBP1) and accelerate the degradation of FBP1, so as to promote the glycolysis and proliferation of pancreatic cancer cells [72].

LDH is the key enzyme that catalyzes the forward reaction of pyruvate reduction to lactate, and LDHB is a subunit of LDH. The serine/threonine kinase Aurora-A can directly bind to lactate dehydrogenase B (LDHB) and phosphorylate it at serine 162. Phosphorylated LDHB reduces the inhibition of substrates in the reduction reaction, significantly accelerates the conversion of pyruvate to lactate, and promotes the glycolysis and proliferation of tumor cells [73]. In addition to LDHB, LDHA, as a component of LDH, also plays an important role in tumor proliferation. In thyroid cancer, lncRNA LINC00671 is negatively correlated with LDHA levels. Under hypoxia condition, the transcription factor STAT3 is activated, which inhibits the expression of LINC00671. This increases the LDHA content in tumor cells and promotes glycolysis and proliferation of thyroid cancer [74].

Fructose-2,6-diphosphatase 3 (PFKFB3) is a regulating enzyme in glycolysis. Under the effect of LINC00930, more RBBP5-GCN5 complexes combine with PFKFB3 promoter. Therefore, H3K4 trimethylation and H3K9 acetylation in the PFKFB3 promoter region increased, resulting in a significant increase in the expression of PFKFB3. Over-expressed PFKFB3 can catalyze the production of lactate, regulate the expression of cell cycle related proteins, and promote glycolysis and proliferation of nasopharyngeal carcinoma cells [75].

As a key enzyme in the glycolysis process, PGK1 catalyzes the conversion of 1,3-bisphosphoglycerate to 3-phosphoglycerate and produces a molecule of ATP. In colorectal cancer, the O-linked N-acetylglucosamine (O-GlcNAc) in PGK1 modifies the glycosylation site at threonine 255. The activity of PGK1 modified by O-GlcNAcylation has been greatly improved and can enter the mitochondria with the help of TOM20 (a translocation enzyme on the outer membrane of mitochondria). PKG1 entering mitochondria can inhibit OXPHOS in cancer cells, but improve glycolysis activity and lactate production. Therefore, colon cancer cells promote tumor proliferation by increasing the level of PGK1 O-GlcNAcylation [76].

ENO1 can catalyze 2-Phosphoglycerate to produce phosphoenolpyruvate. Studies have shown that the overexpression of CD47 is associated with poor prognosis of colorectal cancer. Mechanically, CD47 prevents ENO1 from ubiquitin-mediated degradation by inhibiting FBXW7. This can enhance the glycolysis of tumor cells, activate the ERK signal pathway and ultimately accelerate the proliferation of tumor cells [77]. In lung adenocarcinoma, circ-ENO1 up-regulates the expression of ENO1 gene by interacting with miR-22-3p, resulting in an increase in ATP level, glucose uptake and lactate production of tumor cells, and promoting tumor proliferation [78].

### Glycolysis and tumor angiogenesis and lymphangiogenesis

Tumor angiogenesis and lymphangiogenesis is a complicated process. Abnormality in this process is one of the important characteristics of tumor metastasis [79]. Dickkopf associated protein 2 (DKK2) can cooperate with lipoprotein receptor-related protein 6 (LRP6) and activate downstream AKT/mTOR signal pathway to accelerate aerobic glycolysis of colorectal cancer cells. At this time, a large amount of lactate produced by glycolysis accumulates in the tumor microenvironment, further stimulating the growth of endothelial cells, accelerating tumor angiogenesis and metastasis of colorectal cancer [80].

Tumor lymphangiogenesis and metastasis to sentinel lymph node are critical for tumor progression. Li et al. found that the expression of S100A4 in mice can enhance the movement ability of lymphatic endothelial cells (LECs) and promote lymphangiogenesis and lymph node metastasis of melanoma by studying the mouse model of melanoma popliteal lymph node metastasis. Under hypoxic conditions, S100A4 expression in LECs is up-regulated, which activates AMPK-dependent glycolysis, and regulates the budding of tumor lymphatic vessels by affecting the position and movement of tip cells in the new lymphatic vessels [81].

### Glycolysis and tumor metastasis

In the tumor microenvironment, cancer-associated fibroblasts (CAF) are a large number of stromal cells that can secrete a variety of cancer cell regulatory factors [82]. The regulatory factors secreted by CAF can promote the genesis and development of tumor. CAF also has strong glycolysis ability and releases a large amount of lactate outside the cell. After that, this part of lactate will be absorbed by the cancer cells, converted into pyruvate in the cancer cell plasma, and then provide continuous energy for the proliferation of cancer cells through the OXPHOS of mitochondria. This process is called “reverse Warburg effect” [83]. Ataxia-telangiectasia mutant protein kinase (ATM) in cancer-associated fibroblasts can be induced to oxidize under hypoxia. Oxidized ATM can phosphorylate GLUT1 and increase PKM2 expression to promote glycolysis. In this process, a large amount of accumulated lactate can activate TGFβ1/p38 MAPK/MMP2/9 signal axis and stimulate the mitochondrial activity of cancer cells to improve the invasion and metastasis ability of breast cancer cells [84].

The animal model by Li et al. showed that circRPN2 inhibits glycolysis and metastasis of hepatocellular carcinoma. Specifically, circRPN2 inhibits aerobic glycolysis of HCC by accelerating ENO1 degradation and regulating the miR-183-5p/FOXO1 axis, ultimately inhibiting liver cancer metastasis [85]. In addition, Zhou and colleagues analyzed data from the TCGA database and found that the ZEB1-PFKM-glycolysis axis was significantly associated with tumorigenesis and intrahepatic metastasis of HCC. Moreover, the same results were obtained in the follow-up experiment of *in-situ* HCC xenograft assays [86].

### Glycolysis and tumor immune escape

Tumor “immune escape” refers to the phenomenon that cancer cells escape the surveillance and attack of the immune system, so as to survive and proliferate in the body [87]. Considerable evidences show that tumor glycolysis is strongly related to immune escape of various cancer cells [88, 89].

It has been found that the positive feedback loop formed by Notch1 signaling and TAZ has the effect of promoting the expression of glycolytic genes and accelerating lactate production in cancer cells. Lactic acid accumulated in the extracellular fluid can inhibit T and NK cell activity and induce immune escape from lung cancer [90]. In breast cancer, increased glycolysis activity can up regulate IL-17 signaling pathway, recruit macrophages and other immune cells, but reduce the aggregation of tumor killer cells. High glycolysis can also induce the expression of immune checkpoints such as PD-L1 and CTLA-4, so that breast cancer can escape the surveillance of the immune system [91]. Furthermore, tumor glycolysis can also promote the expression of granulocyte colony-stimulating factor (G-CSF) and granulocyte macrophage colony-stimulating factor (GM-CSF) in triple-negative breast cancer (TNBC) cells, induce the generation of tumor derived myeloid-derived suppressor cells (MDSCs), and affect tumor immune escape and tumor growth [92].

In glioblastoma, high levels of aerobic glycolysis transfer HK2 from mitochondria to cytoplasm. Then, HK2 acts as a protein kinase to phosphorylate IκBα and induce PD-L1 expression, leading to tumor immune escape [93]. Moreover, it has been found that tumor-associated macrophages (TAM) participate in the glycolysis and immune escape of non-small cell lung cancer by reducing the oxygen content in the tumor immune microenvironment, secreting TNF to promote tumor glycolysis, and inhibiting the expression of PD-L1 in tumor cells [94].

### Glycolysis and tumor prognosis evaluation

18 F-FDG PET/CT imaging is a non-invasive tool for evaluating the stage, recurrence or treatment response of various malignant tumors [95, 96]. The most commonly used imaging agent for this examination is 18 F-FDG, which is a glucose analogue. After being injected into the human body, it can enter the cells like glucose and be phosphorylated to form FDG-6-phosphate. However, it cannot be further metabolized and can only accumulate in cells. Tumor cells are characterized by increased glucose metabolism, which is manifested by increased glucose uptake and metabolic rate. Therefore, the imaging principle of PET/CT is to use the high metabolism, high uptake and accumulation of 18 F-FDG in various malignant tumors to find the primary and metastatic lesions.

At present, 18 F-FDG PET/CT parameters that combine tumor volume and tumor metabolic activity, such as metabolic tumor volume and total lesion glycolysis, are prognostic imaging biomarkers for predicting multiple cancers, such as oropharyngeal squamous cell carcinoma [97], epithelial ovarian carcinoma [98], renal cell carcinoma [99] and colorectal cancer [100].

### Glycolysis and tumor treatment

In order to adapt to the harsh tumor microenvironment, tumor cells provide energy support for their various life activities by regulating aerobic glycolysis. Therefore, the destruction of tumor cell glycolysis is considered as a potential tumor treatment strategy. At present, several inhibitors targeting glycolysis have been developed, but further research is still needed to make them play a more comprehensive role in tumor inhibition [101].

DHNQ is a new inhibitor of phosphatidylinositol 3-kinase (PI3K) signal pathway. DHNQ blocks the glycolysis of cancer cells by reducing the expression of glycolytic enzyme genes such as PFK1 and PKM2, so as to inhibit the proliferation, migration and angiogenesis of colorectal cancer [102]. In castration-resistant prostate cancer (CRPC) cells, androgen receptors directly bind to the promoter of the GLUT1 gene, promoting GLUT1 transcription to meet the large glucose demand of CRPC. Inhibition of GLUT1 can significantly reduce the glycolysis and proliferation rate of CRPC cells, make CRPC more sensitive to the treatment of enzalutamide and reduce drug resistance [103].

Metformin is a drug that selectively inhibits the activity of HK2. It can destroy the glycolysis of tumor cells by activating AMP-AMPK pathway and p53 pathway. Glucose oxidase reduces the content of glucose in tumor tissue by consuming accumulated glucose, which improves the efficacy of starvation treatment. The combination of the two drugs has better tumor inhibition effect than the single drug [104].

In addition to the inhibitors mentioned above, there are also some natural compounds that can regulate tumor glycolysis and proliferation. Xu et al. found that chrysin, a bio active flavone, slowed down the glycolysis rate of liver cancer cells and accelerated the apoptosis of liver cancer cells by reducing the expression of HK2 [105]. Piperlongumine is another natural compound with anti-tumor effects. In non-small cell lung cancer, Piperlongumine can reduce the expression of HK2, inhibit the glycolysis of cancer cells, and accelerate cancer cell apoptosis to achieve anti-tumor effects [106].

## m6A regulates tumor glycolysis

**Fig. 3:**
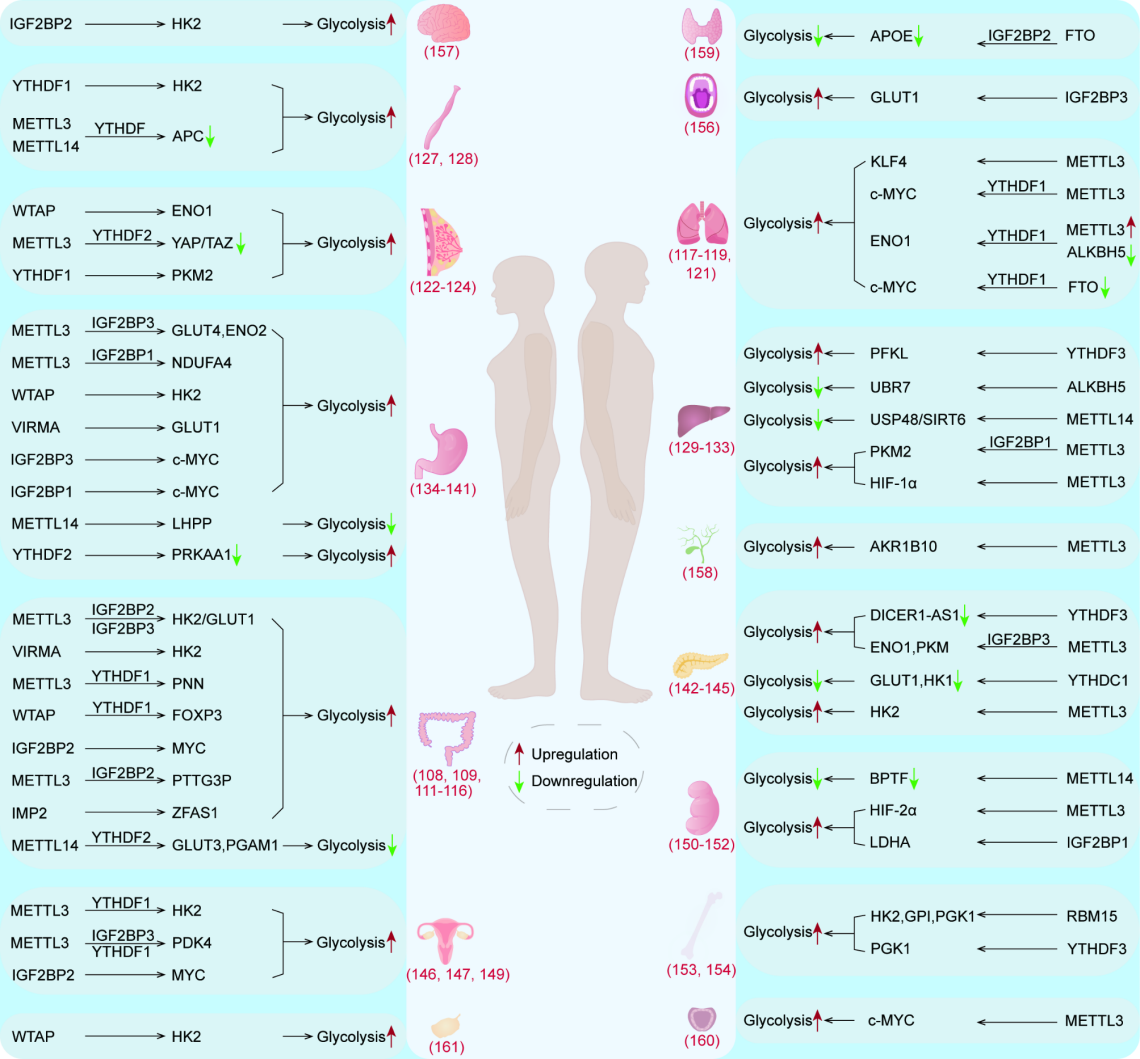
m6A regulates tumor glycolysis. Writers, erasers and readers of m6A participate in tumor glycolysis by regulating glycolysis related enzymes or pathways in a series of direct or indirect ways, and ultimately affect the genesis and development of tumors

Mounting evidence suggests that m6A modification regulates tumor glycolysis through a variety of ways, which is of great significance for tumor proliferation, metastasis and treatment (Fig. 3; Table 3).

**Table 3:** m6A methylation in tumor glycolysis

Cancer type	Regulator	Function	Result	Ref
Colorectal cancer	METTL3, IGF2BP2, IGF2BP3	METTL3, IGF2BP2 and IGF2BP3 jointly promote the expression of HK2 and GLUT1	Improve the glycolysis and proliferation of CRC	[108]
	VIRMA	VIRMA up-regulates the level of HK2 mRNA and improves its mRNA stability	Accelerate glycolysis and induce malignant phenotype of CRC	[109]
	METTL3, YTHDF1	PNN mRNA undergoes m6A modification under the action of METTL3 and improves stability by binding to YTHDF1	Promoting glycolysis and proliferation of colon cancer	[111]
	WTAP, YTHDF1	WTAP and YTHDF1 enhance the stability of FOXP3 mRNA in an m6A-dependent manner	Promoting glycolysis and proliferation of colon adenocarcinoma	[112]
	IGF2BP2	LINRIS influences tumor glycolysis by modulating the IGF2BP2/MYC axis	Improve the glycolysis and proliferation of CRC	[113]
	METTL3, IGF2BP2	PTTG3P is methylated under the action of METTL3 and combines with IGF2BP2 to obtain higher stability	Improve the glycolysis and proliferation of CRC	[114]
	IMP2	IMP2 increases the stability of ZFAS1 RNA in an m6A-dependent manner. In turn, ZFAS1 can enhance the activity of OLA1	Improve the glycolysis of CRC	[115]
	METTL14, YTHDF2	METTL14 and YTHDF2 promote the processing maturity of pri-miR-6769b and pri-miR-499a, and ultimately reduce the expression of GLUT3 and PGAM1	Inhibiting the aerobic glycolysis and malignant phenotype of p53 WT CRC cells	[116]
Lung cancer	METTL3	LncRNA ABHD11-AS1 promotes aerobic glycolysis of NSCLC through ABHD11‐AS1/EZH2/KLF4 axis	Improve the glycolysis of NSCLC	[117]
	METTL3, YTHDF1	DLGAP1-AS2 promotes the expression of c-MYC mRNA in an m6A-dependent manner	Improve the glycolysis and proliferation of lung cancer	[118]
	METTL3, ALKBH5, YTHDF1	METTL3, ALKBH5, and YTHDF1 act on ENO1 mRNA to regulate ENO1 expression	Improve the glycolysis of LUAD	[119]
	FTO, YTHDF1	The decreased expression of FTO can increase the m6A modification in c-MYC mRNA. After that, under the action of YTHDF1, the expression of c-MYC increased	Improve the glycolysis and proliferation of LUAD	[121]
Breast cancer	WTAP	IL1β and TNFα secreted by C5aR1 positive neutrophils can act on the downstream ERK1/2-WTAP-ENO1 signal axis and promote the expression of ENO1	Improve the glycolysis of Breast cancer cells	[122]
	METTL3, YTHDF2	METTL3/LATS1/YTHDF2 axis inhibit the YAP/TAZ axis	Promote glycolysis and tumorigenesis of Breast cancer	[123]
	YTHDF1	Under hypoxia, the transcription of HIF-1α increases, and the expression of PKM2 in cancer cells increases through HIF-1α/miR-16-5p/YTHDF1/PKM2 axis	Improve the genesis and metastasis of Breast cancer	[124]
Esophageal cancer	YTHDF1	HCP5 can enhance the binding of YTHDF1 and m6A-modified HK2 mRNA, thus improving the stability of HK2 mRNA	Improve the glycolysis and malignant phenotype of ESCC	[127]
	METTL3, METTL14, YTHDF	APC mRNA degrades under the joint action of METTL3, METTL14 and YTHDF. Therefore, the regulatory effect of APC on WNT/β-catenin pathway is weakened	Improve the aerobic glycolysis and development of ESCC	[128]
Liver cancer	YTHDF3	YTHDF3 inhibits the degradation of PFKL mRNA by m6A modification	Promote the growth and lung metastasis of HCC	[129]
	ALKBH5	UBR7 activates the Keap1/Nrf2/Bach1/HK2 axis to reduce the content of HK2 in hepatoma cells. Besides, UBR7 expression is regulated by ALKBH5	Inhibit the glycolysis and proliferation of HCC	[130]
	METTL14	Mettl14/USP48/SIRT6 axis has the function of inhibiting liver cancer	Inhibit the glycolysis of HCC	[131]
	METTL3, IGF2BP1	Under the action of METTL3 and IGF2BP1, LNCAROD can increase the level of PKM2 through LNCAROD/PKM2 axis	Induce the glycolysis, proliferation and invasion of HCC	[132]
	METTL3	HBXIP can activate downstream glycolytic enzymes through METTL3/HIF-1α axis	Promote glycolysis and improve the malignancy of HCC	[133]
Gastric cancer	METTL3, IGF2BP3	The expression of HDGF is increased under the action of METTL3 and IGF2BP3. After binding with the promoters of GLUT4 and ENO2, the content of glycolytic enzyme in cells is increased	Promote glycolysis, proliferation and liver metastasis of GC	[134]
	METTL3, IGF2BP1	METTL3 and IGF2BP1 increase the stability of NDUFA4 mRNA and promote the expression of NDUFA4	Improve the glycolysis and proliferation of GC	[135]
	WTAP	WTAP prolongs the half-life of HK2 mRNA	Improve the glycolysis and proliferation of GC	[136]
	VIRMA	LINC00958 degrades less under the action of VIRMA, thus promoting the expression of GLUT1 mRNA	Promote glycolysis of GC	[137]
	IGF2BP3	IGF2BP3 and LIN28B can recognize and bind the m6A site of c-MYC mRNA and promote glycolysis of GC	Promote the proliferation, migration and glycolysis of GC	[138]
	IGF2BP1	The stability of c-MYC mRNA modified with m6A is greatly improved after binding to IGF2BP1 and LIN28B	Promotes the development of gastric cancer	[139]
	METTL14	The stability of LHPP mRNA was enhanced after METTL14-mediated methylation. After that, LHPP inhibited glycolysis of GC by regulating WNT pathway	Inhibit the glycolysis and proliferation of GC	[140]
	FTO, YTHDF2	FTO can reduce the binding of YTHDF2 to the m6A site on PRKAA1 mRNA, increase its stability	Inhibit glycolysis of cancer cells and promote apoptosis	[141]
Pancreatic cancer	YTHDF3	The stability of methylated DICER1-AS1 combined with YTHDF3 decreased, and the ability to promote the maturation of miR-5586-5p decreased. Therefore, the expression of glycolytic genes increases	Promote glycolysis, proliferation and metastasis of pancreatic cancer	[142]
	METTL3, IGF2BP3	Linc-UROD is modified with m6A under the action of METTL3, and its stability is enhanced after binding with IGF2BP3. Linc-UROD can prevent ENO1 and PKM from being degraded by proteasome	Promote the glycolysis and invasion ability of pancreatic cancer	[143]
	YTHDC1	miR-30d is positively correlated with YTHDC1. Besides, miR-30d acts directly with the transcription factor RUNX1 to reduce the expression of GLUT1 and HK1 genes	Inhibit the genesis and glycolysis of pancreatic tumors	[144]
	METTL3	METTL3 promoted the expression of HK2 in an m6A-dependent manner	Promote the glycolysis and PNI of PDAC cells	[145]
Cervical carcinoma	METTL3, YTHDF1	METTL3 modifies HK2 mRNA with m6A at the 3’UTR end of HK2 mRNA, and can also recruit YTHDF1 to combine with HK2 mRNA to improve the stability of HK2 mRNA	Promote the aerobic glycolysis of cervical cancer	[146]
	METTL3, IGF2BP3, YTHDF1	METTL3 can catalyze m6A modification at the 5’UTR of PDK4 mRNA. After that, PDK4 and IGF2BP3 combine to obtain stronger stability, or combine with YTHDF1/eEF-2 complex to improve translation efficiency	Promote glycolysis and proliferation of cancer cells	[147]
	IGF2BP2	E6/E7 protein produced by HPV can promote the expression of IGF2BP2, make it recognize and bind more m6A-MYC mRNA	Promote the proliferation and glycolysis of cervical cancer cells	[149]
Renal carcinoma	METTL14	The transcription factor BPTF can enhance the glycolysis of cancer cells to drive the distant metastasis of renal cell carcinoma. However, this process is inhibited by METTL14	Inhibit the glycolysis and the distant metastasis of renal cell carcinoma	[150]
	METTL3	MTHFD2 has a positive regulatory effect on METTL3-induced HIF-2α mRNA methylation	Promote the glycolysis and proliferation of cancer cells	[151]
	IGF2BP1	IGF2BP1 directly binds to the m6A modification site on LDHA mRNA, thereby enhancing the stability of LDHA mRNA	Promoting glycolysis and malignant phenotype of renal cell carcinoma	[152]
Osteosarcoma	RBM15	Circ-CTNNB1 directly combines with RBM15 to increase the expression of glycolytic enzyme in an m6A-dependent manner	Promote glycolysis and development of osteosarcoma	[153]
	YTHDF3	The stability of m6A-modified PGK1 mRNA was improved after binding to YTHDF3	Promote glycolysis and proliferation of osteosarcoma cells	[154]
Oral squamous cell carcinoma	IGF2BP3	m6A-circFOXK2 and IGF2BP3 act synergistically on GLUT1 mRNA to improve its stability	Promote glycolysis and transfer of OSSC	[156]
Glioblastoma	IGF2BP2	IGF2BP2 can recognize and combine methylated CASC9 to enhance the stability of CASC9. The complex can also improve the stability of HK2 mRNA	Promote glycolysis of GBM	[157]
Cholangiocarcinoma	METTL3	Under the action of METTL3, the stability and expression of AKR1B10 mRNA are improved	Promote the growth and glycolysis of cholangiocarcinoma	[158]
Thyroid papillary carcinoma	FTO, IGF2BP2	APOE induces the expression of glycolytic enzymes through the IL-6/JAK2/STAT3 signal pathway. However, FTO can inhibit the expression of APOE	Inhibit the glycolysis and proliferation of thyroid papillary carcinoma	[159]
Prostate cancer	METTL3	The stability of lncRNA SNHG7 was improved under the action of METTL3. High expression of SNHG7 can activate the downstream SRSF1/c-MYC/glycolytic axis	Promote the glycolysis and development of prostate cancer	[160]
Ovarian cancer	WTAP	In hypoxic environments, the activation of the HIF-1α/WTAP/miR-200/HK2 axis increases	Enhances the glycolysis and proliferation of ovarian cancer	[161]

### Colorectal cancer

Colorectal cancer (CRC) is the second leading cause of cancer death in the world [107]. In colorectal cancer cells, METTL3 directly acts on HK2 and GLUT1 mRNA, and the stability of HK2 and GLUT1 mRNA modified by m6A increases. In the subsequent process, IGF2BP2 combines with 5’/3’UTR regions of HK2. IGF2BP2/3 combine with the 3’UTR region of GLUT1. This promotes the expression of HK2 and GLUT1, and improve the glycolysis and proliferation of colorectal cancer cells [108]. Another methyltransferase VIRMA increases the methylation level of HK2 mRNA in an m6A-dependent manner, up-regulates the level of HK2 mRNA and improves its mRNA stability, which can ultimately accelerate the aerobic glycolysis of cancer cells and improve the degree of malignancy [109]. Liu et al.’s analysis shows that m6A can modify the GLUT1 gene to enhance the stability of GLUT1 mRNA, thereby promoting the glycolysis and cell proliferation of CRC [110]. Pinin (PNN) is a desmosome associated protein that is involved in the development of some tumors. He et al. found that PNN mRNA undergoes m6A modification under the action of METTL3 and improves stability by binding to YTHDF1, ultimately promoting glycolysis and proliferation of colon cancer [111]. Zhang et al. found that WTAP and YTHDF1 enhance the stability of FOXP3 mRNA in an m6A-dependent manner. High expression of FOXP3 can directly promote downstream glycolysis processes, and can also play a role in promoting glycolysis and proliferation of colon adenocarcinoma by increasing the expression of SMARCE1 [112].

In addition to directly regulating the mRNA of enzymes involved in glycolysis, m6A modification can also act on some lncRNAs and miRNAs to regulate tumor glycolysis. Wang et al. found that LINRIS (Long Intergenic Noncoding RNA for IGF2BP2 Stability) was at a high level in colorectal cancer cells and was associated with poor prognosis of patients. LINRIS can induce the K139 ubiquitination of IGF2BP2, thus preventing the degradation of IGF2BP2 by lysosomes, which keeps the content of IGF2BP2 protein at a certain level, and promotes the glycolysis and proliferation of cancer cells through the IGF2BP2/MYC/glycolysis axis [113]. Zheng et al. showed that the expression of lncRNA PTTG3P is closely related to the prognosis of patients with colorectal cancer. PTTG3P was methylated under the action of METTL3 and combined with IGF2BP2 to obtain higher stability. After that, the glycolysis and proliferation of colorectal cancer were improved under the synergistic effect of transcription regulator YAP1 [114]. As the m6A “reader”, IMP2 interacts with the m6A modified lncRNA ZFAS1 through the KH3–4 domain and increases the stability of ZFAS1. The highly expressed ZFAS1 can combine with Obg-like ATPase 1 (OLA1) to improve its ability to hydrolyze ATP and activate glycolysis [115]. Through the study of CRC cells expressing wild-type p53, Hou and his colleagues found that wild-type p53 acts on the promoter region of METTL14 and induces the expression of METTL14. Next, METTL14, with the help of YTHDF2, promoted the biological maturation of pri-miR-6769b and pri-miR-499a. Finally, the miR-6769b-3p/GLUT3 axis and miR-499a-3p/PGAM1 axis can inhibit aerobic glycolysis and malignant phenotype of p53 WT colorectal cancer cells by decreasing the expression of GLUT3 and PGAM1 [116].

### Lung cancer

Lung cancer is the second most common cancer in the world [107]. In non-small cell lung cancer, lncRNA ABHD11-AS1 is more stable after being modified with m6A mediated by METTL3, and promotes aerobic glycolysis of NSCLC through ABHD11‐AS1/EZH2/KLF4 axis [117]. Zhang and his colleagues found that METTL3 can also increase the methylation level of lncRNA DLGAP1-AS2 and improve its stability. Next, DLGAP1-AS2 and YTHDF1 work together to promote the expression of c-MYC mRNA, accelerate the growth of cancer cells and enhance glycolysis [118].

In lung adenocarcinoma (LUAD), ENO1 promotes tumor development in an m6A-dependent manner. The up-regulation of METTL3 and down-regulation of ALKBH5 increased the overall m6A level and ENO1 mRNA methylation level in lung adenocarcinoma. The combination of YTHDF1, a m6A “reader”, with methylated ENO1 mRNA leads to increased expression of ENO1 and promotes glycolysis of lung adenocarcinoma [119]. Liu and colleagues found that NPM1 is associated with m6A modification and glycolysis through analysis of the TCGA and GEO datasets. They also believe that m6A modification may enhance the glycolytic capacity and development of LUAD by enhancing the stability of NPM1 to promote the expression of glycolytic enzymes such as ENO1, HK2, LDHA, LDHB, and GLUT1 in LUAD [120]. Yang et al. showed that the WNT/β-catenin axis can act on the promoter region of FTO to inhibit the expression of FTO. This can increase the modification of m6A in c-MYC mRNA and recruit YTHDF1 to promote the translation of c-MYC mRNA, ultimately accelerating the glycolysis and proliferation of lung adenocarcinoma cells [121].

### Breast cancer

So far, breast cancer has surpassed lung cancer to become the most common cancer in the world [107]. In breast cancer, C5aR1 positive neutrophils can enhance the glycolysis of breast cancer cells. Mechanically, IL1β and TNFα secreted by C5aR1 positive neutrophils can act on the downstream ERK1/2-WTAP-ENO1 signal axis, increase the m6A modification level of ENO1 mRNA, and promote ENO1 expression and glycolysis in breast cancer cells [122]. Xu and his colleagues found that m6A can also regulate glycolysis in breast cancer through Hippo pathway. Specifically, METTL3/LATS1/YTHDF2 axis promotes glycolysis and tumorigenesis of breast cancer by inhibiting YAP/TAZ in Hippo pathway [123]. Yao et al. found that under hypoxia, the transcription of HIF-1α in tumor cells increased, and the expression of miR-16-5p was inhibited, resulting in the decrease of the binding of miR-16-5p and YTHDF1 mRNA, and promoting the expression of YTHDF1. YTHDF1 then binds to PKM2 mRNA to increase PKM2 expression and glycolysis in cancer cells, accelerating the genesis and metastasis of breast cancer [124]. In addition, bioinformatics analysis shows that GPI is closely related to m6A modification in breast cancer and may be a new prognostic marker for breast cancer [125].

### Esophageal cancer

At present, the overall mortality and incidence rate of esophageal cancer rank sixth and seventh respectively [107]. A TCGA ESCA cohort analysis showed that the key glycolytic enzyme HK2 was closely related to m6A modification in esophageal cancer [126]. Functional studies show that HLA complex P5 (HCP5) can enhance the binding of YTHDF1 and m6A-modified HK2 mRNA, thus improving the stability of HK2 mRNA, promoting the aerobic glycolysis of ESCC cells, and increasing the malignant phenotype of ESCC [127].

APC is a common tumor suppressor gene. In esophageal cancer, METTL3 and METTL14 act synergistically on APC mRNA, and the APC mRNA modified by m6A degrades after binding with YTHDF. Therefore, the regulatory effect of APC on WNT/β-catenin pathway is weakened, and the expression of cyclin D1, c-MYC and PKM2 is increased, which promotes the aerobic glycolysis and development of tumor [128].

### Liver cancer

Liver cancer is the sixth most common cancer in the world, but it is the third leading cause of cancer death [107]. In hepatocellular carcinoma, YTHDF3, a m6A “reader”, inhibits the degradation of PFKL mRNA by m6A modification, and promotes the expression of PFKL and aerobic glycolysis. Surprisingly, PFKL positively regulates the expression of YTHDF3 protein, forming a positive feedback loop of YTHDF3-PFKL-YTHDF3, promoting the growth and lung metastasis of liver cancer [129]. The research of Zhao et al. shows that ubiquitin protein ligase E3 component N-recognin 7 (UBR7) activates the Keap1/Nrf2/Bach1/HK2 axis to reduce the content of HK2 in hepatoma cells and inhibit the glycolysis and proliferation of hepatoma cells. However, the m6A “eraser” ALKBH5 promotes the expression of UBR7 in an m6A-dependent manner [130]. Du et al. found that METTL14/USP48/SIRT6 axis also has the function of inhibiting liver cancer. This is because the ubiquitin-specific peptidase 48 (USP48) in hepatoma cells can combine with SIRT6, a histone deacetylase, to improve the stability of SIRT6, which plays a role in weakening the glycolysis of hepatoma cells. USP48 is also regulated by METTL14 [131].

LNCAROD is a lncRNA associated with the glycolysis and poor prognosis of HCC. Under the action of METTL3 and IGF2BP1, the expression and stability of LNCAROD were improved. The high expression of LNCAROD can promote the conversion of PKM to PKM2 mediated by SRSF3, and can also act as a ceRNA of miR-145-5p to maintain PKM2 levels in the cytoplasm. These two pathways synergistically increase the level of PKM2 and induce the aerobic glycolysis, proliferation and invasion of liver cancer cells [132]. Yang and his colleagues found in their research on hepatitis B virus X interacting protein (HBXIP) that the expression of HBXIP can promote glycolysis of liver cancer cells and improve the malignancy. This is because HBXIP can induce the expression of methyltransferase METTL3. In HCC cells overexpressed with METTL3, the m6A modification level of HIF-1α increased, activating downstream glycolytic enzymes and increasing the invasive ability of HCC [133].

### Gastric cancer

The mortality and incidence rate of gastric cancer are the fourth and fifth respectively [107]. In gastric cancer, HDGF mRNA was modified by m6A under the action of METTL3, and its stability and expression increased after binding with IGF2BP3. HDGF in the nucleus can combine with the promoters of GLUT4 and ENO2, increase the content of glycolytic enzymes in cells, and promote glycolysis, proliferation and liver metastasis of gastric cancer [134]. METTL3 can also catalyze m6A modification at the 3’UTR of NADH dehydrogenase-1α sub-complex 4 (NDUFA4) mRNA and recruit IGF2BP1 to increase the stability of NDUFA4 mRNA and promote NDUFA4 expression. As a component of mitochondrial electron transfer chain complex, NDUFA4 promotes the proliferation of gastric cancer cells and tumor growth by promoting glycolysis of GC cells [135]. WTAP, another methyltransferase, catalyzes m6A modification at 3’UTR of HK2 mRNA, prolongs the half-life of HK2 mRNA, and promotes glycolysis and proliferation of GC cells [136].

LncRNA and WNT pathway also play an important role in glycolysis of gastric cancer. For example, LINC00958 can promote aerobic glycolysis of GC cells. In mechanism, m6A methyltransferase VIRMA induced m6A modification on the methylation site of LINC00958. The degradation of LINC00958 was reduced after modification with m6A, and it combined with GLUT1 mRNA to improve the stability of GLUT1 mRNA, thus accelerating the aerobic glycolysis of GC [137]. Xu et al. found that the RNA-binding proteins IGF2BP3 and LIN28B can recognize and bind the m6A site of c-MYC mRNA. LncRNA LOC101929709 acts as a scaffold to support the combination of the above three. The overexpression of c-MYC in turn promotes the expression of LOC101929709 and LIN28B and forms a positive feedback loop that promotes the proliferation, migration and glycolysis of gastric cancer [138]. Besides, Luo et al. found that overexpression of IGF2BP1 is associated with poor prognosis in gastric cancer. Mechanically, the stability of c-MYC mRNA modified with m6A is greatly improved after binding to IGF2BP1. Overexpression of c-MYC promotes the development of gastric cancer through the c-MYC/glycolysis axis [139].

Different from the way described above, the stability of phospholysine phosphohistidine inorganic pyrophosphate phosphotase (LHPP) mRNA is enhanced after METTL14-mediated methylation and plays a role in inhibiting the glycolysis of gastric cancer. After that, the acetylation of LHPP can inhibit the phosphorylation of GSK3b and then inhibit the WNT pathway, so as to inhibit the glycolysis and proliferation of gastric cancer cells [140]. In another study by Zhang et al., it was found that FTO removes the m6A modification in the 3’UTR region of AMPK catalytic subunit α1 (PRKAA1) mRNA, preventing YTHDF2 from recognizing and binding to PRKAA1 mRNA. As a result, the stability of PRKAA1 mRNA increases, ultimately promoting glycolysis in gastric cancer cells and inhibiting apoptosis [141].

### Pancreatic cancer

Pancreatic cancer is a highly malignant tumor, and its death toll is almost the same as the number of patients [107]. In pancreatic cancer, lncRNA DICER1-AS1 can inhibit glycolysis and proliferation of pancreatic cancer. By identifying the m6A modification site on DICER1-AS1, YTHDF3 binds to and destabilizes DICER1-AS1, which reduces maturation of miR-5586-5p. Therefore, the expression of glycolytic genes such as LDHA, HK2, PGK1 and GLUT1 increases, promoting glycolysis, proliferation and metastasis of pancreatic cancer [142]. Another lncRNA linc-UROD was modified with m6A under the action of METTL3, and its stability was enhanced after binding with IGF2BP3. Linc-UROD can prevent ENO1 and PKM from being degraded by proteasome by reducing the ubiquitination of ENO1 and PKM proteins, and finally enhance the glycolysis and invasion ability of pancreatic cancer [143]. miR-30d is a kind of miRNA related to glycolysis and is positively correlated with YTHDC1. miR-30d acts directly with the transcription factor RUNX1 to reduce the expression of GLUT1 and HK1 genes, and inhibits the genesis of pancreatic tumors by inhibiting aerobic glycolysis [144]. These reports collectively suggest that alterations in RNA m6A modifications, mediated by “writers” and “erasers”, can modulate RNA stability by influencing the binding of different “readers” to m6A-modified RNA, and finally impact tumor glycolysis through downstream pathways.

In addition, Li et al. found that glycolysis of pancreatic ductal adenocarcinoma (PDAC) can promote its perineural invasion (PNI). PNI is a special way of cancer metastasis, which can make PDAC spread to the distance along the nerve fibers. This is because glutamate secreted by nerve cells can bind to NMDAR receptors on PDAC cells, causing calcium influx, and promoting the expression of METTL3 through the downstream Ca^2+^ dependent protein kinase CaMKII/ERK-MAPK pathway. Later, METTL3 promoted the expression of HK2 in an m6A-dependent manner, and enhanced the glycolysis and transfer of PDAC cells [145].

### Cervical carcinoma

Cervical cancer is the fourth most common cancer and the fourth leading cause of cancer death in women [107]. At present, there are many studies on the glycolysis of cervical cancer by METTL3. METTL3 modifies HK2 mRNA with m6A at the 3’UTR of HK2 mRNA, and also recruit YTHDF1 to combine with HK2 mRNA to improve the stability of HK2 mRNA and promote the aerobic glycolysis of cervical cancer [146]. METTL3 can also catalyze m6A modification at the 5’UTR of PDK4 mRNA. After that, PDK4 and IGF2BP3 combine to obtain stronger stability, or combine with YTHDF1/eEF-2 complex to improve translation efficiency and ultimately promote glycolysis and proliferation of cancer cells [147].

Persistent infection of human papillomavirus type 16 and 18 (HPV16/18) is considered to be a high-risk factor for cervical cancer [148]. The E6/E7 protein produced by HPV promotes the expression of IGF2BP2, allowing it to recognize and bind more m6A-MYC mRNA to promote the proliferation and aerobic glycolysis of cervical cancer cells [149].

### Renal carcinoma

In renal cell carcinoma, the transcription factor BPTF can enhance the glycolysis of cancer cells to drive the distant metastasis of renal cell carcinoma. However, this process is inhibited by METTL14. The stability and expression of BPTF mRNA catalyzed by METTL14 decreased. The enhancer action of low levels of BPTF is weakened, leading to reduced activation of downstream enolase 2 and other glycolytic related enzymes, and ultimately, the glycolytic level of renal cell carcinoma is reduced and distant metastasis is inhibited [150]. There is also a mitochondrial enzyme called MTHFD2 in renal cell carcinoma, which has a positive regulatory effect on METTL3-induced HIF-2α mRNA methylation. The expression of m6A modified HIF-2α mRNA increased, which accelerated the aerobic glycolysis and proliferation of cancer cells [151]. In addition, Yuan et al. found that the expression of m6A “reader” IGF2BP1 in renal clear cell carcinoma cells increased compared with normal renal histiocyte. The highly expressed IGF2BP1 directly binds to the m6A modification site on LDHA mRNA, thereby enhancing the stability of LDHA mRNA and promoting glycolysis and malignant phenotype of clear cell renal cell carcinoma [152].

### Osteosarcoma

In osteosarcoma, circ-CTNNB1 directly binds to RBM15 and modifies the mRNA of glycolytic enzymes by m6A. The expression of methylated glycolytic enzymes such as HK2, GPI and PGK1 increased to promote glycolysis and osteosarcoma development [153]. Liu and colleagues found that the stability of m6A-modified PGK1 mRNA was improved after binding to YTHDF3. Afterwards, high expression of PGK1 can promote glycolysis and proliferation of osteosarcoma cells [154]. Besides, Bi et al. analyzed seven prognostic m6A related lncRNAs in osteosarcoma and found that they play an important role in the glycolysis process of osteosarcoma [155].

### Other tumors

Oral squamous cell carcinoma (OSCC) is the most common malignant tumor in the head and neck. The expression of circular RNA circFOXK2 was significantly increased after m6A modification, and was related to the malignant degree of OSCC. In mechanism, m6A-circFOXK2 and IGF2BP3 act synergistically on GLUT1 mRNA to improve its stability and promote glycolysis and transfer of OSSC [156]. In glioblastoma (GBM), lncRNA CASC9 can promote glycolysis and is related to the poor prognosis of GBM. The m6A “reader” IGF2BP2 can recognize and combine methylated CASC9 to enhance the stability of CASC9. The complex can also bind to the methylation site on HK2 mRNA to improve the stability of HK2 mRNA and promote GBM glycolysis [157]. In cholangiocarcinoma, AKR1B10 in aldosterone reductase family is associated with glycolysis and malignant phenotype of cancer cells. Under the action of METTL3, the stability and expression of AKR1B10 mRNA were improved, which promoted the growth and glycolysis of cholangiocarcinoma [158]. In thyroid papillary carcinoma, apolipoprotein E (APOE) induces the expression of GLUT1 and other glycolytic enzymes through the IL-6/JAK2/STAT3 signal pathway to promote the genesis and development of cancer. However, FTO can erase the m6A modification level of APOE mRNA, reduce its binding with IGF2BP2 and reduce its stability, and ultimately inhibit the glycolysis and proliferation of thyroid papillary carcinoma [159]. In prostate cancer, lncRNA SNHG7 is modified with m6A under the action of METTL3 and its stability is improved. High expression SNHG7 can recruit SRSF1, which can improve the stability of c-MYC mRNA and ultimately promote the glycolysis and development of prostate cancer [160]. In ovarian cancer, HIF-1α can promote the expression of WTAP in hypoxic environments. WTAP not only contributes to the recognition of pri-miR-200 by DGCR8, a key component of the canonical microprocessor complex of microRNA biogenesis, but also induces m6A methylation of pri-miR-200 to promote its maturation. Finally, miR-200 enhances the glycolysis and proliferation of ovarian cancer by promoting the expression of HK2 [161].

## Clinical significance of m6A-regulated tumors glycolysis

**Fig. 4:**
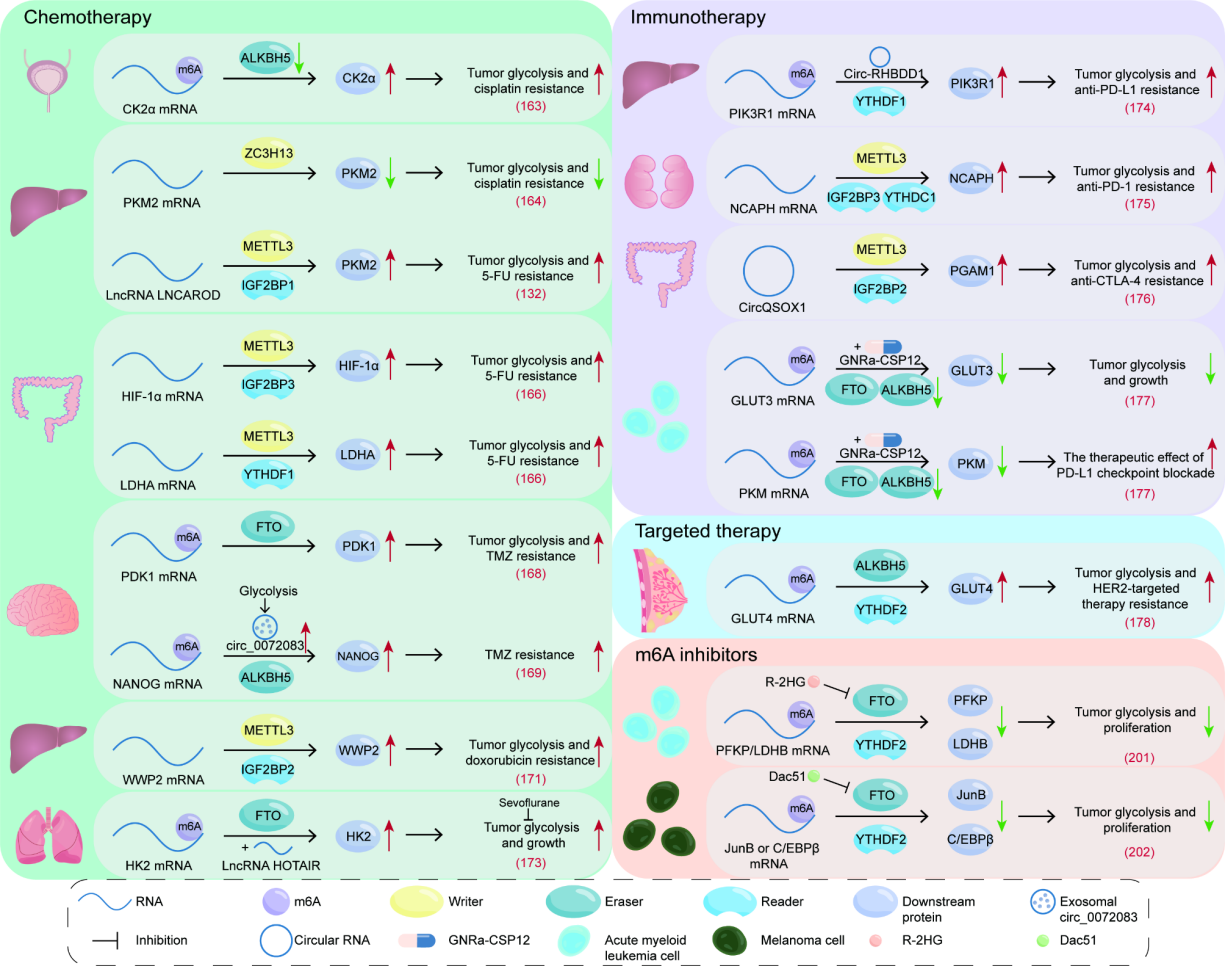
Clinical significance of m6A-regulated tumors glycolysis. m6A regulation impacts the efficacy of tumor chemotherapy, immunotherapy, and targeted therapy by influencing glycolysis. Additionally, m6A inhibitors exert anti-tumor effects by inhibiting glycolysis

The aforementioned information demonstrates that m6A-regulated tumor glycolysis plays an important role in tumor genesis and development. This also aroused our curiosity about whether targeting m6A-regulated tumor glycolysis is beneficial to tumor treatment. Therefore, here, we further discuss the mechanism of m6A-regulated tumor glycolysis in cancer pathogenesis, drug treatment response and drug resistance, and the therapeutic potential of targeting m6A-regulated tumor glycolysis (Fig. 4; Table 4).

**Table 4:** m6A affects tumor therapy by regulating tumor glycolysis

Classification	Regulator	Function	Result	Ref
Chemotherapy	ALKBH5	The low expression of ALKBH5 cannot reduce the m6A modification level of CK2α mRNA, which increases the stability of CK2α mRNA, prolongs its half-life	Reduce the sensitivity of bladder cancer cells to cisplatin chemotherapy	[163]
	ZC3H13	The m6A modification induced by methyltransferase ZC3H13 can reduce the stability of PKM2 mRNA, inhibit the glycolysis of liver cancer cells	Make HCC more sensitive to cisplatin treatment	[164]
	METTL3, IGF2BP1	Under the action of METTL3 and IGF2BP1, LNCAROD can promote the glycolysis and malignancy of HCC	Induce HCC resistance to 5-FU	[132]
	METTL3, IGF3BP3, YTHDF1	METTL3 can induce HIF-1α mRNA methylation and recruit IGF3BP3 to improve its stability, thus improving the transcription level of LDHA. METTL3 also catalyzes m6A modification in the CDS region of LDHA mRNA and recruits YTHDF1 to bind with m6A-LDHA mRNA	Induce CRC resistance to 5-FU	[166]
	FTO	JPX can directly bind to PDK1 mRNA and can also enhance FTO-mediated demethylation of PDK1 mRNA, both of which improve the stability of PDK1 mRNA	Induce GBM resistance to TMZ	[168]
	ALKBH5	Aerobic glycolysis induces the release of exosomal circ_0072083, thus promoting the expression of NANGO mediated by ALKBH5	Induce GBM resistance to TMZ	[169]
	METTL3, IGF2BP2	Under the action of METTL3 and IGF2BP2, the expression of WWP2 increases and promotes liver cancer glycolysis through the WWP2/AKT axis	Induce HCC resistance to doxorubicin	[171]
	FTO	LncRNA HOTAIR can collaborate with FTO to remove the methylation modification of HK2 mRNA, thereby increasing the expression of HK2	Sevoflurane can inhibit HOTAIR and FTO mediated promotion of liver cancer glycolysis	[173]
Immunotherapy	YTHDF1	CircRHBDD1 can recruit YTHDF1 and combine with m6A modified PIK3R1 mRNA, improve the translation level of PIK3R1 mRNA, promote the glycolysis of liver cancer cells through subsequent PI3K/AKT signal transduction pathway	Make HCC resistant to anti-PD-L1 treatment	[174]
	METTL3, IGF2BP3, YTHDC1	During the expression of NCAPH, IGF2BP3 and YTHDC1 regulate the stability of NCAPH mRNA and nuclear output in an m6A-dependent manner. The highly expressed NCAPH can promote the glycolysis of cancer cells and increase the expression of PD-L1 by stabilizing the β-catenin protein	Induce resistance to anti-PD-1 therapy	[175]
	METTL3, IGF2BP2	m6A modified circQSOX1 can recruit miR-326 and miR-330-5p to increase the expression of PGAM1, enhance the glycolysis of CRC and increase the production of lactate	Reduce the anti-CTLA-4 therapeutic effect	[176]
	FTO, ALKBH5	GNRa-CSP12 can destroy the balance of intracellular Fe^2+^/Fe^3+^, inactivate the Fe^2+^-dependent demethylase FTO and ALKBH5. This can reduce the stability of GLUT3 and PKM transcripts that mediate glycolysis and immune checkpoint pathway	Enhance the therapeutic effect of PD-L1 checkpoint blockade	[177]
Targeted therapy	ALKBH5, YTHDF2	ALKBH5 removes the m6A modification of GLUT4 mRNA in cancer cells, and recruits YTHDF2 to combine with GLUT4 mRNA to enhance its stability, leading to increased glycolysis of breast cancer cells	Induce the resistance of breast cancer to HER2 targeted therapy	[178]
m6A inhibitor	FTO	R-2HG competitively inhibits the enzymatic activity of FTO, reduces the stability and expression of PFKP and LDHB transcripts	Inhibit glycolysis and hinder the proliferation of AML	[201]
	FTO	Dac51 effectively inhibits the enzymatic activity of FTO and reduces the stability and expression of JunB and C/EBPβ transcripts	Inhibit glycolysis and hinder the proliferation of melanoma	[202]

### In tumor chemotherapy

Chemotherapy of cancer is a method of treating cancer by using certain chemical drugs to destroy or inhibit the proliferation of cancer cells.

Some studies have shown that the m6A modification in tumor glycolysis is related to the response to chemotherapy. Cisplatin can inhibit the division of cancer cells and induce cancer cell death. Therefore, it has become an effective chemotherapy drug for the treatment of multiple solid tumors [162]. In bladder cancer tissues, the low expression of ALKBH5 cannot reduce the m6A modification level of CK2α mRNA, which increases the stability of CK2α mRNA, prolongs its half-life, ultimately accelerates the glycolysis, proliferation, migration and invasion of bladder cancer cells, and reduces the sensitivity of bladder cancer cells to cisplatin chemotherapy [163]. Wang et al. found in the study of liver cancer that the m6A modification induced by methyltransferase ZC3H13 can reduce the stability of PKM2 mRNA, inhibit the glycolysis of liver cancer cells and reduce the degree of malignancy, and also make liver cancer more sensitive to cisplatin treatment [164].

5-FU is produced by replacing hydrogen with fluorine at C-5 position of uracil, and exerts its anti-tumor effect by inhibiting thymidylate synthase [165]. Jia et al. found that LNCAROD can promote the glycolysis and malignancy of HCC and induce the resistance of HCC to 5-FU in an m6A-dependent manner [132]. In colorectal cancer, METTL3 can induce HIF-1α mRNA methylation and recruit IGF3BP3 to improve its stability, resulting in an increase in the transcription level of LDHA; On the other hand, METTL3 catalyzes m6A modification in the CDS region of LDHA mRNA and recruits YTHDF1 to bind with m6A-LDHA mRNA. Through the above two pathways, the expression of LDHA in colorectal cancer cells increased significantly, and the rate of glycolysis increased, which also led to the resistance of CRC cells to 5-FU [166].

Temozolomide (TMZ) is a first-line chemotherapy drug for GBM, which can effectively penetrate the blood-tumor barrier of GBM, and play an anti-tumor role by inducing DNA double-strand breaks in cancer cells [167]. Li et al. found that the high expression of long non-coding RNA just proximally to the X-inactive specific transcript (JPX) can promote the aerobic glycolysis of GBM cells and is related to the chemoresistance of GBM to temozolomide. In GBM cells, JPX can directly bind to PDK1 mRNA and can also enhance FTO-mediated demethylation of PDK1 mRNA, both of which improve the stability of PDK1 mRNA. In summary, JPX promote GBM aerobic glycolysis to accelerate tumor development and TMZ resistance in an m6A-dependent manner [168]. Other studies have shown that aerobic glycolysis induces the release of exosomal circ_0072083, thus promoting the expression of NANGO mediated by ALKBH5 and making GBM resistant to TMZ [169].

Doxorubicin is a commonly used drug in transcatheter arterial chemoembolization, with broad-spectrum anti-tumor activity [170]. WW domain containing protein 2 (WWP2) is a ubiquitin E3 ligase. The study by Qin et al. suggests that WWP2 regulates glycolysis and proliferation of liver cancer cells in an m6A-dependent manner, and is also associated with the resistance of liver cancer to doxorubicin therapy. Mechanically, the stability of WWP2 mRNA was improved under the combined action of METTL3 and IGF2BP2. Overexpression of WWP2 inhibits the anti-liver cancer effect of doxorubicin through WWP2/AKT/glycolysis axis [171].

Numerous evidence suggests that sevoflurane can inhibit the growth of cancer cells and inhibit platelet induced invasion of cancer cells [172]. Sun et al. found that the stability of HK2 mRNA in lung cancer cell lines decreased after m6A modification. However, lncRNA HOTAIR can collaborate with FTO to remove the methylation modification of HK2 mRNA, thereby increasing the expression of HK2 and promoting glycolysis and proliferation of lung cancer. This process is inhibited by sevoflurane [173].

### In tumor immunotherapy

Tumor immunotherapy is to restore the normal anti-tumor immune response of the body by restarting and maintaining the tumor-immune cycle. At present, the effect of m6A modified tumor glycolysis on the treatment of tumor immune checkpoints inhibitors (anti-PD-L1/anti-PD-1 and anti-CTLA-4) has attracted people’s attention.

In liver cancer, circRHBDD1 can recruit YTHDF1 and combine with m6A modified PIK3R1 mRNA, improve the translation level of PIK3R1 mRNA, promote the glycolysis of liver cancer cells through subsequent PI3K/AKT signal transduction pathway and make liver cancer resistant to anti-PD-1 treatment. Therefore, the combined treatment of glycolytic inhibitors and immunosuppressants for liver cancer deserves our attention [174]. Chen et al. found that non-smc lectin I complex subunit H (NCAPH) has a strong correlation with glycolysis and immune tolerance of clear cell renal cell carcinoma. During the expression of NCAPH, IGF2BP3 and YTHDC1 regulate the stability and nuclear export of NCAPH mRNA in an m6A-dependent manner. The highly expressed NCAPH can promote the glycolysis of cancer cells, increase the expression of PD-L1 and induce resistance to anti-PD-1 therapy by stabilizing the β-catenin protein [175]. In colorectal cancer, circQSOX1 is methylated under the action of METTL3, and its stability is improved after binding with IGF2BP2. m6A modified circQSOX1 can recruit miR-326 and miR-330-5p to increase the expression of PGAM1, enhance the glycolysis of CRC, increase the production of lactate, promote the immune escape of CRC and reduce the therapeutic effect of anti-CTLA-4. It can be seen that the combined treatment of sh-circQSOX1 and anti-CTLA-4 is of great value in avoiding the drug resistance of CRC immunotherapy [176]. In addition, GNRa-CSP12 is a potential immunosuppressive agent for the treatment of leukemia. It can destroy the balance of intracellular Fe^2+^/Fe^3+^, inactivate the Fe^2+^-dependent demethylase FTO and ALKBH5, and increase the level of intracellular m6A. This can reduce the stability of GLUT3 and PKM transcripts that respectively mediate glycolysis and immune checkpoint pathway, so as to inhibit the proliferation of AML cells and enhance the therapeutic effect of PD-L1 checkpoint blockade [177].

### In tumor-targeted therapy

In addition to chemotherapy and immunotherapy, m6A modifications may reduce the efficacy of HER2-targeted therapies for breast cancer by modulating glycolysis. Specifically, ALKBH5 removes the m6A modification of GLUT4 mRNA in cancer cells, and recruits YTHDF2 to combine with GLUT4 mRNA to enhance its stability, leading to increased glycolysis of breast cancer cells and resistance to HER2 targeted therapy. Therefore, inhibiting the ALKBH5/GLUT4 axis is of great significance in improving the efficacy of HER2 targeted therapy for breast cancer, including trastuzumab and lapatinib [178].

### Combining m6A and glycolysis inhibitors for tumor therapy

As mentioned above, tumor glycolysis regulated by m6A has an immeasurable role in chemotherapy, immunotherapy, and targeted therapy of tumors. It also raises the question of whether the combination of m6A and glycolysis inhibitors could contribute to better anti-tumor therapy.

In order to fulfill the glucose demand, the vast majority of cancer cells have a high expression of GLUT1 on the cell membrane. GLUT1 specific inhibitors, such as STF-31 and WZB117, have been developed to block glucose uptake by cancer cells, thereby inhibiting tumor growth [179, 180]. Additionally, several inhibitors targeting key enzymes in the glycolysis process have been investigated, including 2-deoxy-D-glucose (2-DG), PFK158, 3-BrPA, Shikonin, and Galloflavin. These inhibitors act on HK2, PFKFB3, GAPDH, PKM2, and LDH, respectively, effectively inhibiting glycolysis and tumor proliferation [181–186]. Among them, 2-DG, a glycolysis inhibitor targeting HK2, has advanced to clinical trials.

With the advancement of high-throughput screening technology and continuous exploration of the molecular mechanisms underlying m6A modification, several m6A regulatory inhibitors with anti-cancer potential have emerged. For instance, STM2457 and Elvitegravir are specific inhibitors of METTL3, while CWI1-2 and JX5 selectively inhibit IGF2BP2 [187–190]. BTYNB functions as an inhibitor of IGF2BP1, and MV1035 targets ALKBH5 [191, 192]. However, the most significant results have been observed with FTO inhibitors. Rhein, FB23, FB23-2, MO-I-500, MA2, CS1 and CS2 have all demonstrated promising anti-tumor effects by inhibiting FTO’s m6A demethylase activity [193–200]. Notably, we highlight two FTO inhibitors, R-2-hydroxyglutarate (R-2HG) and Dac51. R-2HG competitively inhibits FTO’s enzymatic activity, resulting in increased m6A modifications on PFKP and LDHB mRNA in AML cells. Subsequently, YTHDF2 identifies and binds to these transcripts, reducing their stability and expression, and ultimately hindering AML proliferation by inhibiting the glycolytic pathway [201]. In melanoma, Dac51 enhances m6A modification levels in JunB and C/EBPβ mRNA by inhibiting FTO, which facilitates the binding of YTHDF2 to both mRNAs, leading to significant reductions in JunB and C/EBPβ expression. JunB and C/EBPβ are transcription factors that activate genes involved in glycolysis, such as PFKP, PGAM1, and HK1, thereby promoting glycolysis in cancer cells and limiting the activation and effector states of CD8^+^ T cells, promoting tumorigenesis and progression. By targeting these processes, the FTO inhibitor Dac51 effectively suppresses melanoma. Interestingly, overexpression of JunB in FTO-KD cells was sufficient to inhibit CD8^+^ T cell activation. Blocking tumor glycolysis with the HK2 inhibitor 2-DG significantly restored impaired CD8^+^ T cell activation [202].

The available evidence strongly suggests that m6A can modulate the sensitivity of various cancer cell types to anti-cancer therapies by regulating tumor glycolysis. Therefore, the combination of m6A inhibitors with tumor glycolysis inhibitors holds promise in enhancing anti-tumor efficacy. We eagerly anticipate early breakthroughs in the clinical application of these inhibitors, as they bring hope for the future of cancer treatment.

## Conclusion and future perspectives

The story between tumor and glycolysis has been explored for decades. The most typical example is the discovery of Warburg effect, which reveals differences between tumor cells and normal cells from the perspective of glucose metabolism. With the rapid development of m6A detection technologies such as MeRIP-seq, miCLIP and SELECT-m6A, there are more and more studies on the relationship between m6A and tumor glycolysis [203–205]. We found that m6A modification regulates glycolysis and proliferation of tumor cells through a series of direct or indirect ways. For example, m6A can directly modify the mRNA of key glycolysis enzymes such as HK2, GLUT1, ENO1 and PDK4, and regulate tumor glycolysis by influencing the expression of these enzymes [108, 119, 147]. m6A can also modulate lncRNA and circRNA to regulate tumor glycolysis by controlling their downstream pathway. Examples include the circFOXK2/IGF2BP3/GLUT1 axis in OSSC and the lncRNA CASC9/IGF2BP2/HK2 axis in glioblastoma [156, 157]. From the current research, we can predict that m6A regulates tumor glycolysis through an extremely complex interaction network, and thereby affects the genesis, development and treatment of tumor. Further study of the potential link between m6A and tumor glycolysis will help us gain a deeper understanding of tumor metabolism and have important implications for the future development of new tumor diagnostic methods and treatment options.

Although the strategy of targeting m6A-modified tumor glycolysis for tumor therapy shows great promise, there are still challenges in related research. As early as the 1950s, m6A modification was detected in organisms, but the research on m6A regulation of tumor glycolysis began to be carried out in recent years [206]. Therefore, the relevant research is still in the initial stage, and there are many potential mechanisms that have not been discovered. From a metabolic point of view, the heterogeneity of tumor cells makes different cells in tumor tissues have different metabolic states, which poses a serious challenge for targeted glycolysis in cancer therapy [207]. Currently, preclinical studies have demonstrated the anti-tumor properties of certain glycolytic inhibitors, such as metformin and natural compounds chrysin and piperlongumine [104–106]. Besides, in vitro and animal studies have shown that inhibiting METTL3 in colorectal cancer cells can reduce glycolysis levels and restore therapeutic sensitivity to 5-FU [166]. In drug-resistant breast cancer cells, specific knockdown of ALKBH5 inhibited glycolysis and restored therapeutic response to trastuzumab and lapatinib in vitro and in vivo experiments [178]. It can be predicted that the combined action of m6A inhibitors and tumor glycolysis inhibitors will open a new window of hope for the treatment of tumors in the future. However, the existing specific drugs and small molecule inhibitors of m6A regulators and tumor glycolysis are in the development stage, and have not yet entered the clinical trial stage. There is an urgent need to identify specific inhibitors that have strong anti-tumor efficacy, high bioavailability and few drug-related side effects in order to be applied in clinic as soon as possible [71, 200, 208, 209].

In summary, glucose metabolism is closely related to tumor proliferation, angiogenesis and lymphangiogenesis, metastasis, immune escape, prognostic evaluation and treatment. In different types of tumors, m6A modification affects the biological behavior, therapeutic response and prognosis of tumors by altering the mRNA stability of key glycolytic enzymes and activating or inhibiting glycolytic-related signaling pathways. Therefore, further exploration of the complex relationship and mechanism of m6A methylation regulation of tumor glycolysis provides a new idea for future tumor therapy. Developing specific inhibitors targeting m6A regulators and tumor glycolysis will be a new direction for tumor treatment in the future.

## Data Availability

Not applicable.

## Abbreviations

ALKBH: ALKB homolog
APOE: Apolipoprotein E
ATM: Ataxia-telangiectasia mutant protein kinase
ATP: Adenosine-triphosphate
CAFs: Cancer-associated fibroblasts
CCCH: Cys-Cys-Cys-His
CRC: Colorectal cancer
CRPC: Castration-resistant state cancer
DKK2: Dickkopf associated protein 2
ENO: Enolase
FBP1: Fructose-1,6-diphosphatase
FTO: Fat mass and obesity-associated protein
GAPDH: Glyceraldehyde-3-phosphate dehydrogenase
GLUT: Glucose transporters
GPI: Glucose-6-phosphate isomerase
G-CSF: Granulocyte colony-stimulating factor
GM-CSF: Granulocyte macrophage colony-stimulating factor
HBXIP: Hepatitis B virus X interacting protein
HCP5: HLA complex P5
HNRNP: Heterogeneous nuclear ribonucleoprotein
HNRNPA2B1: Heterogeneous nuclear ribonucleoprotein A2/B1
HK: Hexokinase
JPX: Just proximally to the X-inactive specific transcript
LDH: Lactate dehydrogenase
LECs: Lymphatic endothelial cells
LHPP: Phospholysine phosphohistidine inorganic pyrophosphate phosphotase
LINRIS: Long Intergenic Noncoding RNA for IGF2BP2 Stability
LRP6: Receptor-related protein 6
LUAD: Lung adenocarcinoma
m6A: n6-methyladenosine
MAT2A: Methionine adenosyltransferase 2 A
MDSCs: Myeloid-derived suppressor cells
METTL: Methyltransferase Like
NCAPH: Non-smc lectin I complex subunit H
NDUFA4: NADH dehydrogenase-1α sub-complex 4
NKAP: NF-κB activating protein
OLA1: Obg-like ATPase 1
OSCC: Oral squamous cell carcinoma
O-GlcNAc: O-linked N-acetylglucosamine
OXPHOS: Oxidative phosphorylation
PDAC: Pancreatic ductal adenocarcinoma
PFK: Phosphofructokinase-1
PFKFB3: Fructose-2,6-diphosphatase 3
PGAM: Phosphoglycerate mutase
PGK: Phosphoglycerate kinase
PI3K: Phosphatidylinositol 3-kinase
PK: Pyruvate kinase
PNI: Perineural invasion
RBM15: RNA binding motif protein 15
SAM: S-adenosylmethionine
SFPQ: Splicing factor, proline- and glutamine-rich
TMZ: Temozolomide
TNBC: Triple-negative breast cancer
UBR7: Ubiquitin protein ligase E3 component N-recognin 7
USP48: Ubiquitin-specific peptidase 48
UTR: Untranslated region
VCRs: Vertebrate-conserved regions
VIRMA: Vir like m6A methyltransferase associated protein
WTAP: Wilms tumor 1-associated protein
YTHDC: YTH Domain Containing
YTHDF: YT521-B homology Domain-containing Family
ZC3H13: Zinc finger CCCH-type containing 13
ZCCHC4: Zinc finger CCHC-type containing 4
